# Transcriptome Analysis Identifies ALCAM Overexpression as a Prognosis Biomarker in Laryngeal Squamous Cell Carcinoma

**DOI:** 10.3390/cancers12020470

**Published:** 2020-02-18

**Authors:** Pedro Nicolau-Neto, Paulo Thiago de Souza-Santos, Mariana Severo Ramundo, Priscila Valverde, Ivanir Martins, Izabella Costa Santos, Fernando Dias, Tatiana de Almeida Simão, Luis Felipe Ribeiro Pinto

**Affiliations:** 1 Programa de Carcinogênese Molecular, Instituto Nacional de Câncer—INCA, Rua Andre Cavalcanti 37, Rio de Janeiro, RJ CEP 20231-050, Brazil; pedronicolau.n@gmail.com (P.N.-N.); marianasevero@gmail.com (M.S.R.); 2 Laboratório de Hanseníase, Instituto Oswaldo Cruz—Fiocruz, Av. Brasil, 4365 - Manguinhos, Rio de Janeiro, RJ CEP 21040-900, Brazil; pthiagoss@gmail.com; 3 Divisão de Patologia, Instituto Nacional de Câncer—INCA, Rua Cordeiro da Graça, 156, Rio de Janeiro, RJ CEP 20220-400, Brazil; pvalverde@inca.gov.br (P.V.); ioliveira21@hotmail.com (I.M.); 4 Seção de Cirurgia de Cabeça e Pescoço, Instituto Nacional de Câncer—INCA, Praça da Cruz Vermelha, Rio de Janeiro, RJ CEP 20230130, Brazil; ibellacs@yahoo.com.br (I.C.S.); fdiasmd@yahoo.com.br (F.D.); 5 Departamento de Bioquímica, IBRAG, Universidade do Estado do Rio de Janeiro, Av. 28 de Setembro 87, Fundos, Pavilhão Américo Piquet Carneiro-4º andar, Rio de Janeiro, RJ CEP 20551-030, Brazil; tasimao@gmail.com

**Keywords:** laryngeal squamous cell carcinoma, transcriptome analysis, prognostic biomarker, ALCAM

## Abstract

Background: Laryngeal squamous cell carcinoma (LSCC) is one of the most incident tumors in the world, especially in developing countries, such as Brazil. Different from other tumors, LSCC prognosis did not improve during the past four decades. Therefore, the objective of this study was to develop biomarkers that can predict LSCC patient’s prognosis. Results: Transcriptome analysis pointed out 287 overexpressed genes in LSCC in comparison to adjacent mucosa. Among these, a gene-pattern signature was created with 24 genes associated with prognosis. The Bayesian clustering of both Brazil and The Cancer Genome Atlas (TCGA) data pointed out clusters of samples possessing significative differences in the prognosis, and the expression panel of three genes (*ALCAM*, *GBP6*, and *ME1*) was capable to distinguish patients with worse prognosis with an accuracy of 97%. Survival analyses with TCGA data highlighted *ALCAM* gene expression as an independent prognostic factor for LSCC. This was further confirmed through immunohistochemistry, using a validation set of Brazilian patients. *ALCAM* expression was not associated with prognosis for other head and neck tumor sites. Conclusion: ALCAM overexpression seems to be an independent prognosis biomarker for LSCC patients.

## 1. Introduction

Laryngeal squamous cell carcinoma (LSCC) is a highly incident and mortal disease [1], affecting mainly the male population of medium- and low-income countries, such as Brazil that presents the fourth highest incidence of this disease in the world [2,3]. LSCC diagnosis and treatment is multidisciplinary, with the employment of different procedures. Nevertheless, 60% of patients present advanced disease, and LSCC is one of few tumors with decreasing five-year survival rates over the past 40 years [4,5]. Consequently, there is a demand to improve therapy response. 

The application of molecular biomarkers in the diagnosis, prognosis, and treatment choice was essential to reduce the mortality rates of prostate, breast, colorectal, and lung cancer [6,7,8,9]. Recently, The Cancer Genome Atlas (TCGA) consortium published a comprehensive molecular study on head and neck squamous cell carcinoma (HNSCC) [10]. However, not only several molecular alterations were not site-specific, but also, they were not analyzed regarding clinicopathological features [11]. There is a lack of a more comprehensive molecular analyses concerning LSCC prognosis [5], resulting in gene-specific or small gene panel analyses. So, at the somatic level, mutations in *CDKN2A* and *TP53*, and copy number alterations in *CDKN2A*, *PIK3CA*, and *HER2* were associated with worse survival rates of patients with LSCC. Besides, analysis of mutations in *CDKN2A* and *TP53* in laryngeal dysplasia could predict lesions that would develop into a tumor [12,13,14]. In addition, hypermethylation of *CDKN2A* gene body was associated with better locoregional control after surgery [15], and *LMX1B* hypermethylation was associated with worse overall and disease-free survival rates [16]. Additionally, the expression of long noncoding RNAs, such as *CCAT1*, *DGR5*, *H19*, and *HOTAIR*, were also associated with LSCC prognosis and diagnosis [17]. The signature of claudin expression, specifically claudin 1, 3, 7, and 8, was associated with early diagnosis and metastasis identification and related to prognosis [18], whereas the analysis of tumor-associated immune cells components, especially CD3, CD4, CD8, CD68, and CD163 positive cells, was described as useful to predict the response of immunological checkpoint inhibitor therapy [19].

Therefore, the objective of this study was to identify biomarkers associated with LSCC prognosis. To achieve it, we carried out a transcriptomic analysis and validated the identified genes in our own validation set of samples, and also in the TCGA database, revealing ALCAM overexpression as an independent prognostic factor for LSCC patients.

## 2. Results

### 2.1. Identifying Molecular Prognostic Biomarker for LSCC Patients 

Transcriptome analysis revealed 725 differentially expressed genes (DEG), 287 overexpressed and 438 underexpressed, in LSCC when compared to nonmalignant surrounding mucosa (NSM) (Table S1). These DEGs were related to cell signaling pathways associated with neoplastic progression, such as cell-extracellular matrix interaction, focal adhesion, PI3K/AKT, and small cell lung cancer-associated pathway. 

**Figure 1:**
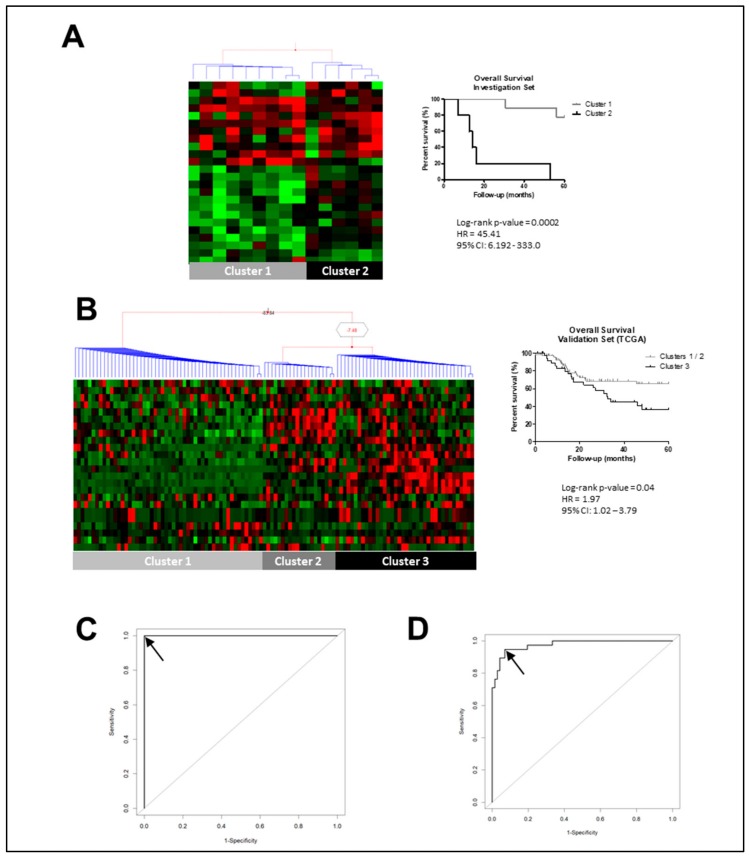
Laryngeal squamous cell carcinoma (LSCC) transcriptome analysis pointed out the gene expression signature associated with prognosis. (**A**) Bayesian hierarchical clustering with the expression of gene-pattern signature associated with LSCC prognosis was capable to segregate Brazilian National Cancer Institute (INCA) LSCC samples into two clusters. Kaplan–Meier curve analysis shows prognosis differences between LSCC samples according to gene-expression signature, with Cluster 1 samples presenting a significative better prognosis than Cluster 2 samples. (**B**) Applying the gene-prognosis panel to The Cancer Genome Atlas (TCGA) LSCC samples revealed three clusters from Bayesian hierarchical clustering, and Cluster 3 presented a worse prognosis than samples from Clusters 1 and 2. In the heatmaps, each column represents an individual sample and each line represents a gene expression. The red and green colors represent increased and decreased gene expression, respectively. Groups were made according to 24 overexpressed gene expressions associated with prognosis. (**C**) The 12-gene-expression panel (*ADH7*, *ALCAM*, *CYP2C19*, *GBP6*, *LYPD6B*, *TPD52L1*, *ODC1*, *BTBD11*, *PTGR1*, *ME1*, *C12ORF75*, and *ACVR1*) was capable to distinguish samples of Cluster 3, which presented worse prognosis, from those from Clusters 1 and 2 with an AUC of 1.0. (**D**) A three-gene panel with *ALCAM*, *GBP6*, and *ME1* expression values showed similar results to those from 12-gene panel, showing AUC of 0.97, sensitivity 94.7%, and specificity 93.1%. Legend: HR, hazard ratio; CI, confidence interval; black arrow represents the selected ROC curve point.

Among the 287 overexpressed genes, 24 were associated with prognosis (log-rank *p*-value < 0.05), creating a suggestive prognostic gene-pattern expression signature panel of LSCC (Table 1). Therefore, the expression value of this prognostic gene-pattern signature was used in clustering LSCC samples in the investigation set of samples, resulting in two groups with significative differences in prognosis. Cluster 1 contained samples from patients who presented better prognosis (median survival 129.4 months) than patients grouped in Cluster 2 (median survival 14.10 months) (*p* = 0.0002, Harzard Ratio (HR) = 45.41, 95% confidence interval (CI) = 6.19−333.0) (Figure 1A). Applying this gene set to TCGA data, three clusters of LSCC samples were observed. Cluster 3 showed a five-year survival rate of 36.3%, presenting a significative worse prognosis than samples from Clusters 1 and 2, which possessed a five-year survival rate of 65.4% (Figure 1B). 

The ROC (receiving operating characteristic) curve analysis with all 24 genes applied to TCGA validation set revealed area under curve (AUC) of 1.0 to detect LSCC patients with worse prognosis, and the same result was observed when using as a minimal subset of 12 genes (*ADH7*, *ALCAM*, *CYP2C19*, *GBP6*, *LYPD6B*, *TPD52L1*, *ODC1*, *BTBD11*, *PTGR1*, *ME1*, *C12ORF75*, and *ACVR1*). Further analysis reducing the number of genes from the panel revealed that with three genes (*ALCAM*, *GBP6*, and *ME1*) we could reach an accuracy of 0.97 (sensitivity of 94.7% and specificity of 93.1%). Further reduction in the number of genes caused a significant loss of accuracy (Figure 1C,D).

**Figure 2:**
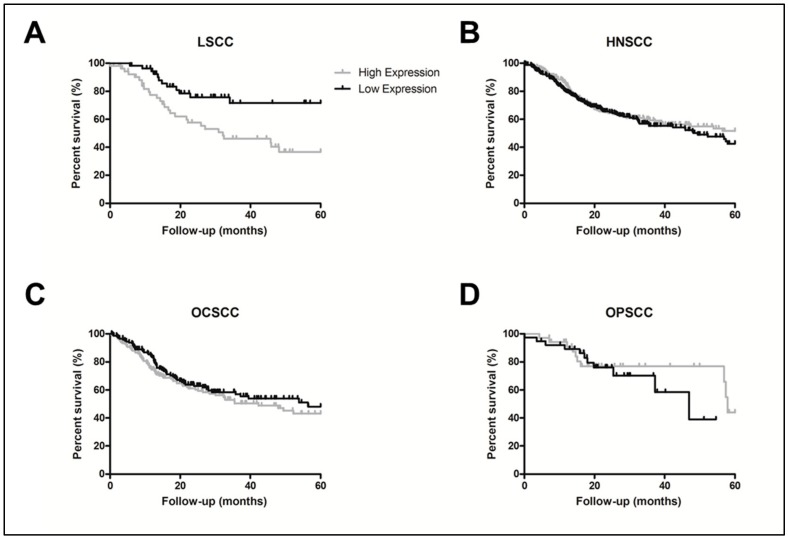
*ALCAM* overexpression confers worse prognosis to LSCC patients. (**A**) LSCC presenting *ALCAM* low expression have a better prognosis than LSCC overexpressing *ALCAM* (*p* = 0.01, HR = 2.74, 95% CI 1.26–5.97). The association with prognosis seems to be specific to LSCC among HNSCC tumors. *ALCAM* expression was not associated with prognosis analyzing all head and neck squamous cell carcinoma (HNSCC) together (**B**) or separately, oral cavity (OCSCC) (**C**) and oropharnyngeal (OPSCC) (**D**). Legend: Black line, *ALCAM* low expression; grey line, *ALCAM* high expression. Groups were made according to *ALCAM* expression median value.

To validate the prognostic value of each individual gene present in the gene panel, we conducted univariate survival analyses using LSCC data from TCGA, with *ALCAM* (*p* = 0.01), *BTBD11* (*p* = 0.20), *LOX* (*p* = 0.04), and *LYPD6B* (*p* = 0.16) being maintained for Cox regression multivariate analysis. Final Cox regression model showed involved surgical margins (*p* = 0.001, HR = 4.11, 95% CI = 1.75–9.66), and *ALCAM* expression (*p* = 0.010, HR = 2.74, 95% CI = 1.26–5.97) as independent prognostic factors (Table 2; Figure 2A). The *ALCAM* gene overexpression association with prognosis was exclusive for LSCC in the HNSCC TCGA data, and was not observed either when all HNSCC samples were analyzed together (*p* = 0.97), or according to other specific sites (oral cavity (OCSCC), *p* = 0.34; and oropharyngeal (OPSCC), *p* = 0.36) (Figure 2B–D). 

Aiming to understand possible reasons behind overexpression of *ALCAM*, we also analyzed *ALCAM* somatic alterations in the LSCC TCGA dataset. *ALCAM* was amplified in 11.8% of LSCC samples, showing association with the expression levels (*p* = 0.018). Only one sample showed a missense mutation, with unknown biological significance (Figure S1).

### 2.2. ALCAM Protein High Levels Was also Associated to LSCC Worse Prognosis 

**Figure 3:**
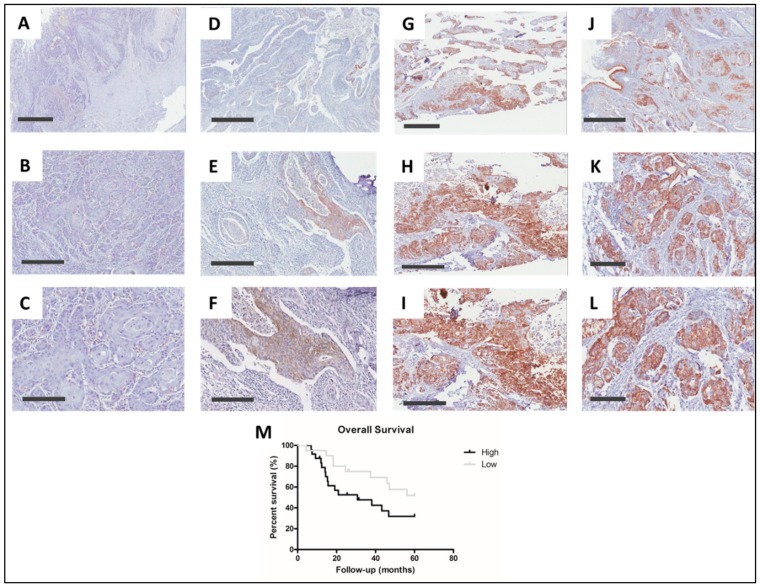
ALCAM protein levels in LSCC and its association to prognosis. Representative micrographs of ALCAM protein expression analysis by immunohistochemistry performed in 44 LSCC. In this analysis, 27.3% of LSCC showed no ALCAM expression (**A**–**C**). Among ALCAM positive tumors, 25% of samples showed expression in less than 20% of cells being categorized as low ALCAM expression (**D**–**F**). LSCC samples with more than 20% of positive cells were categorized as high ALCAM expression (**G**–**L**). (**G**–**I**) Representative LSCC with ALCAM staining in 70% of cells. (**J**–**L**) Representative LSCC with ALCAM staining in 80% of cells. (**M**) LSCC presenting ALCAM protein low levels have a better prognosis than LSCC overexpressing ALCAM protein (*p* = 0.04, HR = 2.31, 95% CI 1.03 – 5.28). (**A**,**D**,**G**,**J**) grey bar represents scale 1 mm. (**B**,**E**,**H**,**K**) grey bar represents scale 400 µm, (**C**,**F**,**I**,**L**) grey bar represents 200 µm. Black line, ALCAM high levels; grey line, ALCAM low levels. Groups were made according to ALCAM median value of positive cells.

In order to validate the association between ALCAM overexpression and LSCC prognosis, we evaluated ALCAM protein expression by immunohistochemistry in 44 LSCC samples (Figure 3A–L). This analysis showed that 12 tumors (27.3%) had no ALCAM expression, while 32 (72.7%) presented positive ALCAM immunostaining. The median percentage of positive staining cells was 20%, and this value was used as cut-off for classifying samples with low or high ALCAM levels. In this way, eight samples (25%) presented low ALCAM levels, and 24 (75%) tumors presented high expression. ALCAM immunostaining was restricted to cell membranes and presented a direct correlation with *ALCAM* gene expression (*r* = 0.37, *p* = 0.029, 95% CI = 0.03–0.63) (Figure S2). ALCAM protein immunohistochemical analysis confirmed the worse prognosis associated with *ALCAM* gene overexpression. Patients with high ALCAM levels presented a lower median survival time (30.7 months) compared to those tumors showing low or negative ALCAM levels (137.9 months), and high ALCAM protein levels was also an independent prognostic factor for LSCC (*p*= 0.04, HR = 2.31, 95% CI = 1.03–5.28) (Figure 3M). No association was observed between ALCAM protein levels and LSCC clinical-pathological characteristics.

## 3. Discussion

LSCC is one of the few tumors that have presented decreasing overall survival rates during the past decades. Therefore, in this manuscript we developed a gene-expression panel that is strongly associated with LSCC patient’s prognosis. Among the 24 genes that made the panel, ALCAM gene and protein expression was shown to be an independent prognostic factor for LSCC.

Activated leukocyte cell adhesion molecule (*ALCAM*) gene is located at human chromosome 3q13.11 [20], and encodes a transmembrane glycoprotein, which acts in the cell–cell adhesion, either in homotypic (ALCAM–ALCAM) or in heterotypic (ALCAM–CD6) interactions between adjacent cells [21]. *ALCAM* expression could be detectable in a variety of tissues and cells under certain spatial and temporal controls during development [14]. In homeostasis, homotypic ALCAM interactions could modulate epithelial and endothelial cells’ interactions and neuronal guidance, while ALCAM–CD6 heterotypic interaction shows physiological relevance in antigen presentation in immune cell adhesion [22,23,24,25]. Several studies show a role for CD6 as a co-stimulatory molecule in T-cell activation [26,27] and the ALCAM–CD6 interaction was described as pivotal for antigen presentation [28,29]. Interestingly, it was observed that the molecule I/F8 scFv induces ALCAM internalization and the conjugation between I/F8 scFv and the saporin immunotoxin efficiently kill ALCAM-positive tumor cells selectively [30]. Additionally, vaccine-induced cytotoxic T-lymphocytes can recognize an epitope expressed by ALCAM and this could be useful as a novel mechanism of induction of potent tumor-specific cellular responses by mimotopes of tumor-associated carbohydrate antigens [31].

ALCAM seems to characterize cancer stem cells (CSC) in some tumors and *ALCAM* was highly expressed in intestinal stem cell niche, with an association to intestinal carcinoma progression, including benign and metastatic tumors [32]. Subpopulation of nonsmall cell lung cancer (NSCLC) triple-positive for EPCAM, ALCAM, and CD44 possessed CSC characteristics, including being highly proliferative, having greater clonogenicity, ability for self-renewal through spheroid formation, and chemoresistance [33]. Recently, ALCAM-E3 ligase-mediated degradation was associated with CSC features’ regulation in HNSCC cells [34]. Besides, ALCAM membrane expression was considered a CSC marker in OCSCC-derived cell lines [35]. We are going to carry out in vitro analysis with LSCC cell lineages to try to understand the role of ALCAM overexpression in LSCC prognosis.

The long arm of chromosome 3, which presents the *ALCAM* gene, is a classically amplified genomic region in squamous carcinomas, particularly in esophageal squamous cell carcinoma and HNSCC [10,11]. However, only 12% of LSCC samples presented ALCAM copy number gain/amplification associated with its overexpression, suggesting that further mechanisms, such as DNA methylation, already shown to be associated with ALCAM overexpression in breast tumors [36], may also be associated with this deregulation in LSCC. 

Although our data pointed out *ALCAM* gene expression association only with the prognosis of LSCC patients among HNSCC, our study was the only one that evaluated this marker in the larynx exclusively. In other HNSCC studies, ALCAM protein overexpression, evaluated by immunohistochemistry, was already related as an independent prognostic factor for OCSCC, associated with the sonic hedgehog signaling pathway [37], or Epidermal Growth Factor Receptor (EGFR) activation [38], in the Chinese population. In a similar way, ALCAM protein level was described as potential biomarkers for predicting tumor behavior and prognosis of salivary gland tumor in Iranian patients [39]. Recently, Clauditz et al. [40] evaluated the ALCAM protein expression in HNSCC, including LSCC samples, combining in the same group of samples laryngeal and hypopharyngeal tumors, and observed a discordant result to our findings, being ALCAM expression mainly cytoplasmic, and not associated with the prognosis of LSCC patients. 

Recently, studies have proposed the potential use of gene-expression signature to measure the prognosis of LSCC patients, employing both protein-coding and -noncoding genes. Concerning protein-coding genes, a panel of 26 hypoxia-related genes was associated with the improvement of hypoxia-modifying treatment in laryngeal cancer [41], and the expression of 18 inflammatory-associated genes was capable to distinguish LSCC samples according to prognosis with AUC of 0.61 [42]. A panel of two long noncoding RNAs was also associated with LSCC prognosis presenting AUC of 0.69 [43]. The limited number of studies that propose biomarkers for LSCC prognosis reflects in only one clinical trial recruiting LSCC patients according to a biomarker, aiming to block PD1/PD-L1 interaction. 

Although our transcriptomic analysis was conducted in a limited number of samples, it was one of the few studies that evaluated exclusively LSCC, separate from the large HNSCC group. Moreover, our data exposed the prognostic value of the gene panel and ALCAM in both Brazilian and TCGA samples. Besides the prognostic value of ALCAM expression measurement, our study pointed out a valuable prognostic gene-expression signature, which shows high power to discriminated LSCC samples regarding their patient’s outcome (12-genes signature AUC 1.00, 3-genes signature ROC 0.97), which can improve the treatment option and patient monitoring, aiming to improve treatment response. 

## 4. Materials and Methods 

### 4.1. LSCC Samples

A total of 44 LSCC and paired nonmalignant surrounding mucosa (NSM, histopathologically adjacent normal mucosa, 3 cm from tumor borders) samples were collected from 2008 to 2014 by the Head and Neck Surgical Division of the Instituto Nacional de Câncer (INCA, Rio de Janeiro, Brazil) from patients who had not undergone chemo- or radiotherapy treatment. Histopathological profiling was evaluated by the Pathology Department of INCA. All patients signed an informed consent form, and the project was approved by the institution’s Ethics Committee. 

Among these set of LSCC and NSM samples, 14 LSCC and 12 NSM samples were randomly selected (investigation set of samples) for transcriptome analysis. The first validation set to confirm gene expression by qPCR and immunohistochemistry analysis were conducted with samples from all 44 patients. A second validation set was composed by the Head and Neck provisional data from TCGA consortium [10]. TCGA data were analyzed with the web-based software cBioPortal [44,45]. Patients’ clinical and pathological features are described in Table 3.

### 4.2. LSCC Gene-Expression Profiling 

RNA of all samples was isolated from frozen tissue with the RNeasy Mini Kit (Qiagen, Inc, Hilden, Germany). RNA of the investigation set of samples was converted to complementary DNA (cDNA) with WT Expression Kit, biotinylated, and applied to GeneChip Human Exon 1.0 ST array (Affymetrix, Inc., Santa Clara, CA, USA), as previously described [46]. The raw data were normalized in the Expression Console software (Affymetrix) using the robust multi-array average (RMA) method. Subsequent analysis of gene expression was carried out in R environment, using the *limma* package, available from the Bioconductor project, to obtain quantitative expression levels for coding genes. Differentially expressed genes (DEG) were classified by the following criteria: *p* < 0.05 and fold-change expression cutoff |2.0|. Microarray data are available at Gene Expression Omnibus Accession Browser (accession number GSE143224) [47,48,49].

### 4.3. Prognostic Gene-Pattern Signature

The prognostic value of all overexpressed genes was evaluated using the microarray data herein performed. For this purpose, we analyzed each gene expression regarding its association with the patient’s prognosis in the investigation set of samples. Higher and lower gene expression were defined, using as cut-off the median expression value. Genes with log-rank *p*-value < 0.05 were used for the Bayesian hierarchical clustering of both investigation and validation set of samples followed by survival analysis between clusters of samples. Receiver-operating characteristic (ROC) curves and the area under the ROC curve (AUC), as well as the sensitivity and specificity values, were used to assess the feasibility of using messenger RNA (mRNA) expression levels as prognostic biomarkers for LSCC patients. Initially, all prognostic-associated gene expressions were included in the ROC curve analysis. Genes were removed from the ROC curve analysis following the backward stepwise method regarding the gene individual AUC value, which resulted in a ROC curve with the lower number of genes possessing significantly high AUC. Survival analyses, Bayesian clustering, and ROC curve analyses were conducted in R using the survival packages, BHC and Epi, respectively [50,51,52].

### 4.4. Gene-Expression Validation by Quantitative PCR

ALCAM expression was assessed by RT-qPCR. The cDNA of 44 paired LSCC and NSM was synthesized with SuperScriptIITM Reverse Transcriptase (Invitrogen^®^) and quantitative PCR was carried out with the Quantifast SYBR Green PCR kit (Qiagen) in a Rotor-Gene 6000 thermal cycler (Qiagen). Gene expression quantification was performed as previously described [53]. Specific oligonucleotides were used in the expression levels analyses, as follows: ALCAM Forward 5′-AAGTGTGCAGTACGACGATGT-3′; ALCAM Reverse 5′-GGTTGCTTGAACACCTTGACT-3′; GAPDH Forward 5′ CAACAGCCTCAAGATCATCAGCAA 3′, GAPDH Reverse 5′ AGTGATGGCATGGACTGTGGTCAT 3′. RT-qPCR analyses were conducted in triplicate, using TE-1 cells as positive control, whereas negative control reactions were performed without cDNA. 

### 4.5. Immunohistochemistry Analysis

Immunohistochemistry (IHC) was performed on 3-μm paraffin sections of all 44 LSCC cases. For ALCAM antigen retrieval, sections were incubated in a steam oven while submerged in a trilogy buffer solution (Cell Marque), for 30 min at 98 °C. Sections were then incubated with the primary monoclonal antibodies against ALCAM (Sigma, St. Louis, MO, USA, HPA010926, working dilution 1:1000), for at least 12 h. Formalin-Fixed Paraffin-Embedded (FFPE) prostate carcinoma samples served as positive control staining. As the negative control, the primary antibody was replaced by the diluent solution. The detection system used was the NovoLinkTM Max Polymer Detection System (Leica Biosystems, Wetzlar, Germany), following the protocol described by the manufacturer, using diaminobenzidine as substrate (Dako). Sections were counterstained with Harris’ hematoxylin. Scored cases were considered positive when at least 1% of epithelial cells were stained. LSCC samples were categorized as low and high ALCAM protein expression using the median number of positive cells as the cut-off. Samples with positive epithelial cells lower than median value were classified as low ALCAM tumors and samples with positive epithelial cells equal or higher than median value were classified as high ALCAM tumors.

### 4.6. ALCAM Somatic Alterations in LSCC

The frequency of ALCAM copy number alterations (CNA) and single nucleotides variants (SNV) in LSCC were evaluated in the LSCC of TCGA using cBioportal software, through whole exome sequencing and DNA microarray applying GISTIC 2.0 protocol [54], respectively. 

### 4.7. Statistical Analyses

Differences in gene expression were evaluated using Kruskal–Wallis test, followed by Dunn’s multiple comparison tests. Spearman’s rank correlation was used for assessing gene and protein expression correlation. All analyses were performed with GraphPad Prism 5 software. In survival analyses using TCGA data, univariate analysis was estimated by the Kaplan–Meier method and log-rank test. Variables with *p* < 0.2 were selected for multivariate analysis. Finally, Cox regression was applied with the stepwise forward method [55]. R environment using the survival package was used for survival analyses. The same protocol of survival analysis was applied for immunohistochemistry data. 

## 5. Conclusions

ALCAM gene and protein expression seems to be an independent prognosis biomarker to LSCC patients.

## Figures and Tables

**Table 1:** Overexpressed genes associated to LSCC prognosis in the validation set of samples.

Gene Symbol	Official Full Name	Log-Rank *p*-Value
***ACOX1***	acyl-CoA oxidase 1	0.007
***ACVR1***	activin A receptor type 1	0.007
***ADH7***	alcohol dehydrogenase 7	0.005
***AGFG2***	ArfGAP with FG repeats 2	0.010
***ALCAM***	activated leukocyte cell adhesion molecule	0.007
***BTBD11***	BTB domain containing 11	0.003
***C12orf75***	chromosome 12 open reading frame 75	0.010
***CDK14***	cyclin dependent kinase 14	0.045
***CYP2C19***	cytochrome P450 family 2 subfamily C member 19	0.005
***GBP6***	guanylate binding protein family member 6	0.045
***GLTP***	glycolipid transfer protein	0.045
***GNG4***	G protein subunit gamma 4	0.010
***LOX***	lysyl oxidase	0.045
***LYPD6B***	LY6/PLAUR domain containing 6B	0.013
***ME1***	malic enzyme 1	0.045
***NPEPPS***	aminopeptidase puromycin sensitive	0.045
***ODC1***	ornithine decarboxylase 1	0.003
***PMM1***	Phosphomannomutase 1	0.016
***PTGR1***	prostaglandin reductase 1	0.000
***SERPINA3***	serpin family A member 3	0.045
***ST3GAL4***	ST3 beta-galactoside alpha-2,3-sialyltransferase 4	0.045
***TPD52L1***	tumor protein D52-like 1	0.045
***ZDHHC13***	zinc finger DHHC-type containing 13	0.010
***ZNF750***	zinc finger protein 750	0.045

**Table 2:** Survival analyses pointed out *ALCAM* gene expression as independent prognostic factor in LSCC.

Feature	Categories	Univariate Analysis			Multivariate Analysis		
		HR	(95% CI)	*p* Value	HR	(95% CI)	*p* Value
LSCC TCGA Provisional Data (*n* = 110)							
Age at diagnosis (years)	>62 vs. <62	1.19	(0.67–2.12)	0.54			
Tumor Stage	III-IV vs. I-II	0.74	(0.31–1.77)	0.51			
Tumor Differentiation	G3 vs. G2 vs. G1	0.69	(0.43–1.12)	0.14	0.98	(0.34–2.82)	0.980
Perineural Invasion	Yes vs. No	3.97	(1.67–9.47)	0.001	2.50	(0.92–6.73)	0.069
Surgical Margins	Positive/Close vs. Negative	4.20	(1.79–9.83)	0.0009	4.11	(1.75–9.66)	0.001
*ACOX1*	High vs. Low	1.04	(0.59–1.85)	0.86			
*ACVR1*	High vs. Low	1.26	(0.70–2.25)	0.43			
*ADH7*	High vs. Low	1.05	(0.59–1.87)	0.84			
*AGFG2*	High vs. Low	0.75	(0.42–1.33)	0.33			
*ALCAM*	High vs. Low	2.05	(1.13–3.69)	0.01	2.74	(1.26–5.97)	0.010
*BTBD11*	High vs. Low	1.44	(0.81–2.54)	0.20	2.26	(0.72–7.06)	0.158
*C12ORF75*	High vs. Low	1.04	(0.59–1.86)	0.87			
*CDK14*	High vs. Low	0.92	(0.51–1.66)	0.78			
*CYP2C19*	High vs. Low	1.23	(0.70–2.18)	0.45			
*GBP6*	High vs. Low	1.09	(0.61–1.94)	0.75			
*GLTP*	High vs. Low	0.90	(0.51–1.61)	0.74			
*GNG4*	High vs. Low	1.44	(0.81–2.54)	0.21			
*LOX*	High vs. Low	1.81	(1.01–3.24)	0.04	1.99	(0.66–6.02)	0.218
*LYPD6B*	High vs. Low	0.65	(0.36–1.18)	0.16	0.60	(0.18–1.95)	0.402
*ME1*	High vs. Low	1.14	(0.64–2.02)	0.64			
*NPEPPS*	High vs. Low	1.16	(0.65–2.05)	0.60			
*ODC1*	High vs. Low	1.38	(0.78–2.46)	0.26			
*PMM1*	High vs. Low	1.21	(0.68–2.15)	0.49			
*PTGR1*	High vs. Low	1.31	(0.74–2.33)	0.34			
*SERPINA3*	High vs. Low	1.44	(0.39–1.23)	0.21			
*ST3GLA4*	High vs. Low	0.80	(0.45–1.43)	0.46			
*TPD52L1*	High vs. Low	0.85	(0.48–1.51)	0.59			
*ZDHHC13*	High vs. Low	0.97	(0.55–1.71)	0.91			
*ZNF750*	High vs. Low	0.90	(0.51–1.60)	0.73			

Footnote: HR, hazard ratio; G1, G2, and G3 represent well, moderately, and poorly differentiated, respectively.

**Table 3:** Clinicopathological data of investigation and validation set of samples.

Feature		Brazilian Samples		Investigation Set		Validation Set			
		(*n* = 44)		Brazilian Transcriptome (*n* = 14)		TCGA Data (*n* = 110)		*p*-Value #	*p*-Value $
		*n*	%	*n*	%	*n*	(%)		
Age (years)	Median	62.5		58		62		0.81	0.43
	Range	44-88		45–77		38–83			
Gender	Male	42	95.45%	13	92.86%	91	82.73%	0.46	0.04
	Female	2	4.55%	1	7.14%	19	17.27%		
	NA	0		0	0.00%	0	0.00%		
Tumor Differentiation	Well	6	13.64%	1	7.14%	7	6.36%	0.25	0.03
	Moderate	34	77.27%	12	85.71%	70	63.64%		
	Poor	4	9.09%	1	7.14%	29	26.36%		
	NA	0		0	0.00%	4	3.64%		
Tumor Stage	I	3	6.82%	1	7.14%	2	1.82%	0.62	1.00
	II	1	2.27%	1	7.14%	9	8.18%		
	III	7	15.91%	2	14.29%	19	17.27%		
	IV	31	70.45%	9	64.29%	80	72.73%		
	NA	2	4.55%	1	7.14%	0	0.00%		
Lymph node metastasis	No	16	36.36%	3	21.43%	39	35.45%	0.35	0.70
	Yes	26	59.09%	9	64.29%	52	47.27%		
	NA	2	4.55%	2	14.29%	19	17.27%		
Perineural Invasion	Negative	29	65.91%	7	50.00%	45	40.91%	1.00	1.00
	Positive	15	34.09%	4	28.57%	24	21.82%		
	NA	2	4.55%	3	21.43%	41	37.27%		
Involved Surgical Margin	Negative	33	75.0%	11	78.57%	81	73.64%	0.43	0.31
	Positive/close	9	20.45%	3	21.43%	13	11.82%		
	NA	2	4.55%	0	0.00%	16	14.55%		
Tobacco Smoking	Current/Former	36	81.82%	10	71.43%	101	91.82%	0.18	0.17
	No	5	11.36%	2	14.29%	6	5.45%		
	NA	3	6.82%	2	14.29%	4	3.64%		
Alcohol Consumption	Current/Former	31	70.45%	6	42.86%	39	35.45%	0.09	1.00
	No	5	11.36%	4	28.57%	7	6.36%		
	NA	8	18.19%	6	42.86%	71	64.55%		

# Feature comparison between Investigation Set and Validation Set. $ Feature comparison between Brazilian Samples and Validation Set.

## Supplementary Materials

The following are available online at https://www.mdpi.com/2072-6694/12/2/470/s1, Figure S1: Somatic alterations in ALCAM gene; Figure S2: ALCAM expression correlation between quantitative PCR (qPCR) and immunohistochemistry (IHC), Table S1: LSCC transcriptome differentially expressed genes list.
