# Supplementary material for: Transcriptome Analysis Identifies ALCAM Overexpression as a Prognosis Biomarker in Laryngeal Squamous Cell Carcinoma
Source: Cancers (Basel). 2020 Feb 18;12(2):470. doi: 10.3390/cancers12020470 (PMC7072229; doi:10.3390/cancers12020470)
Supplement: Supplementary file 1 [file cancers-12-00470-s001.zip › cancers-706732-suppl-final/cancers-706732-suppl-figures.pdf]

Supplementary Material: Transcriptome Analysis Identifies ALCAM Overexpression as a Prognosis Biomarker in Laryngeal Squamous Cell Carcinoma

Pedro Nicolau-Neto, Paulo Thiago de Souza-Santos, Mariana Ramundo, Priscila Valverde, Ivanir Martins, Izabella Costa, Fernando Dias, Tatiana de Almeida Simão and Luis Felipe Ribeiro Pinto

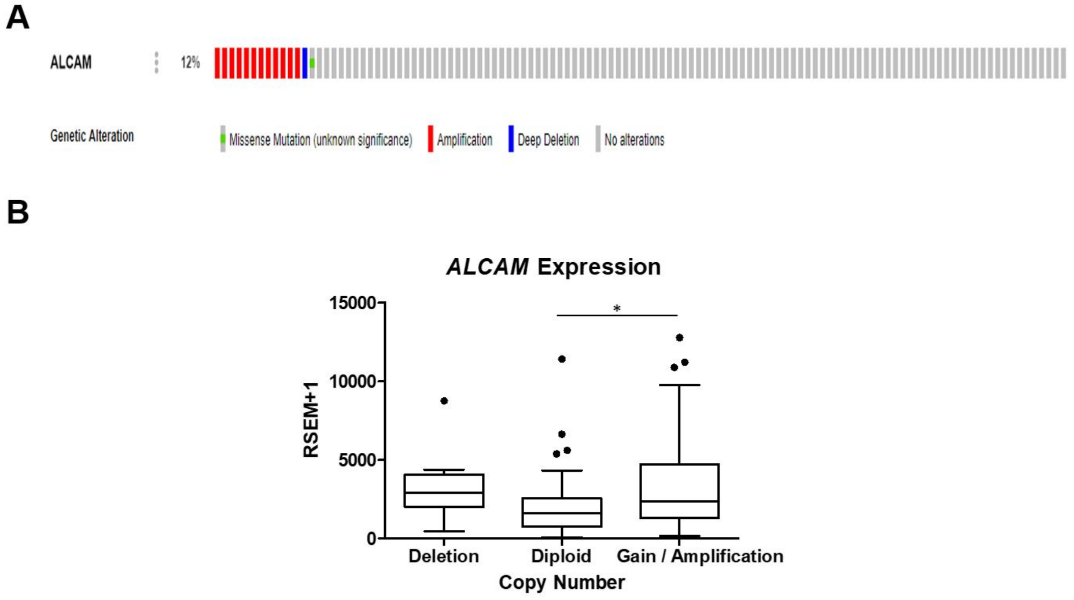

Figure S1. Somatic alterations in ALCAM gene.

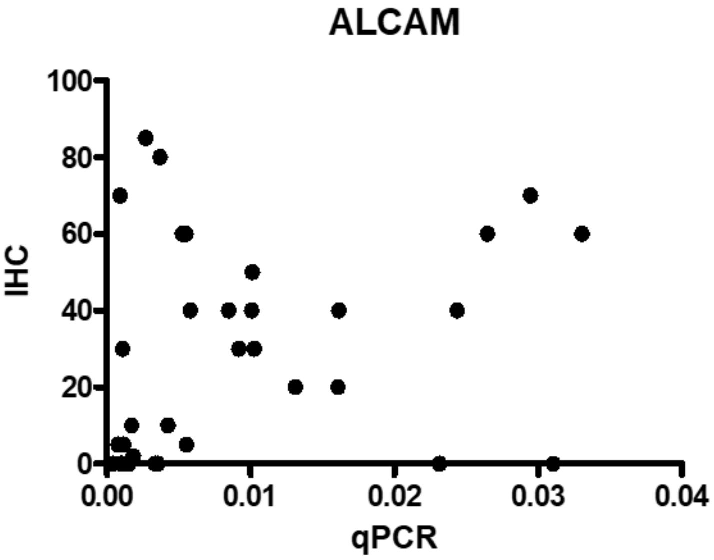

Spearman r	0.3750
95% confidence interval	0.03170 to 0.6392
P value (two-tailed)	0.0289

Figure S2. ALCAM expression correlation between quantitative PCR (qPCR) and immunohistochemistry (IHC)

© 2020 by the authors. Licensee MDPI, Basel, Switzerland. This article is an open access article distributed under the terms and conditions of the Creative Commons Attribution (CC BY) license (http://creativecommons.org/licenses/by/4.0/).
